# Special Provision for Epilepsy

**Published:** 1893-07

**Authors:** 


### EDITORIAL DEPARTMENT.

F. E. DANIEL, M. D., Editor.
S. E. HUDSON, M. D., Managing Editor.
A. J. SMITH. M. D., Galveston, Associate Editor.

EDITORIAL STAFF:

PROF. J. E. THOMPSON, M. D., Texas Medical College, Galveston; Surgery.
PROF. WM. KEILLER, M. D., Texas Medical College, Galveston; Obstetrics and Gynecology.
PROF. DAVID CERNA, M. D., Texas Medical College, Galveston; Therapeutics.
PROF. A. J. SMITH, M. D., Texas Medical College, Galveston; Medicine.
DR. ISADORE DYER, Tulane University; Dermatology.
DR. R. H. L. BIBB, Saltillo, Mexico; Foreign Correspondent.
DR. ROBT. MORRIS, Charity Hospital, N. O.; Clinical Reports.

———o———

The JOURNAL is the official organ of the Austin District Medical Society, the Wes Texas District Medical Society and the Galveston County Medical Society.

### SPECIAL PROVISION FOR EPILEPSY.

Epilepsy is the despair of the general practitioner. The field of medicine is so extensive and so rapidly widening that "specialists" in almost every branch, have become a pressing necessity. No man, especially one remote from medical centers, can keep so thoroughly posted on every branch as to be able, without warning and special preparation, to do full justice to every kind of case that presents. Imagine a man repairing a ruptured perineum, *secumdem artem* at 9 o'clock; extracting a cataract at 10; making a laparotomy at 11; trephining the skull at 12; lancing the baby's gums at 1. Why, he would be more than mortal; and must be super-human indeed, to be able to sandwich between these acts, visits to sick, and to prescribe for diarrhoea, la grippe, cholera morbus, neuralgia and the thousand and one ailments that affect human nature. We want to see learned men narrowing their practice down to one branch, or to one organ, as it *used to be*, in the olden times. Why not? Why not make epilepsy a specialty? There is too much in the general field "neurology;" it ought to be sub-divided. Then, again, all epilepsy is not neurotic, some cases are caused by mechanical pressure, and can be relieved by trephining.

By the bye, this leads us to recall the fact that Dr. B. E.

Hadra, of Galveston, has been creating a stir in surgical circles recently by his investigations upon epilepsy. His paper on "Combined Surgical and Electrical Treatment of the Brain in Epilepsy with Report of Some Cases," which was read by him at the Southern Surgical and Gynecological Association, has awakened a new interest in the subject, and the author has received a great many letters from physicians throughout the country, asking him to continue his investigations and publish the results.

Dr. Hadra is like all learned Germans we ever met; he is skeptical. He will take no man's word for it; will accept nothing which cannot be demonstrated. And that is right. Were there no doubt there would be no inquiry. Were there no inquiry and experiments, there would be no *progress*,—and the world certainly "do more." The JOURNAL feels an interest in Dr. Hadra's investigations, and will watch them with interest. In this connection we will say that during our recent visit to Galveston we visited and inspected the hospitals and college buildings. We were truly surprised at the provision there made for the indigent sick; while for pay patients every luxury and comfort can be had. At the two splendidly equipped hospitals, both under the ablest medical and surgical management, there is ample accommodation; and inquiry elicited the astonishing fact that a patient of limited means can be splendidly cared for and receive the highest skill in treatment, medical or surgical, at an expense not exceeding *one dollar a day*. This covers all expense. Physicians having troublesome cases of epilepsy, can send them to Galveston with the assurance that they will be well cared for and receive the especial attention of Dr. Hadra if a note be dropped him along with the patient.

